# Motivation to Lead: A Study of the Supportive Nursing Leadership Environment

**DOI:** 10.1155/2024/2652746

**Published:** 2024-07-10

**Authors:** Sulaiman Al Sabei, Amy Miner Ross, Asma Al Yahyaei, Leodoro Labrague, Omar Al-Rwajfah, Kylee Deterding

**Affiliations:** ^1^ Fundamentals and Nursing Administration Department College of Nursing Sultan Qaboos University, P.O. Box 50, Al-Khodh 123, Muscat, Oman; ^2^ School of Nursing Oregon Health and Science University, Portland, Oregon, USA; ^3^ School of Nursing and Healthcare Leadership University of Washington, Tacoma, Washington, USA; ^4^ College of Nursing Al Al-Bayt University, Mafraq, Jordan; ^5^ Oregon Health & Science University School of Nursing, Portland, Oregon, USA

## Abstract

**Background:**

With projected nursing shortages, an aging workforce, and the imminent retirement of nurse leaders, nursing leadership shortages are a concern. While several studies have indicated the interest of nurses in pursuing leadership positions, limited research has focused on examining the influence of the leadership practice environment on nurses' motivation to lead.

**Aim:**

The aims of the study were to (1) assess the relationship between the leadership environment and the motivation of nurses to lead and (2) determine whether there are particular aspects of the leadership environment that influence motivation to lead.

**Methods:**

A cross-sectional research design was used to collect data from 435 nurses working in 16 public and private hospitals in Oman. Leadership Environment Scale and Motivation to Lead Scale were used to assess participants' perceived leadership environment and their motivation to engage in formal leadership roles, respectively. Multivariate linear regression was used to assess the relationship between the perceived leadership environment and the motivation of nurses to undertake leadership roles. *Findings*. Nurses reported a mean scale value of 3.208 out of 5 (SD = 0.467) for their motivation to lead, which exceeds the midpoint, indicating a strong motivation to engage in formal leadership roles. Nurses reported a mean score of 3.194 out of 4 (SD = 0.661), which exceeds the midpoint, suggesting a favorable perception of leadership environment. The findings showed a significant relationship between the leadership environment and nurses' motivation to lead. Specifically, self-organization (*β* = 0.185, *p*=0.001, CI = 0.086–0.378), agents (*β* = 0.221, *p*=0.002, CI = 0.124–0.474), and transformative exchange (*β* = 0.100, *p*=0.037, CI = 0.101–0.142) were characteristics of the leadership environment that were associated with greater motivation to engage in leadership.

**Conclusion:**

This study emphasizes the importance of cultivating a supportive leadership environment as a potential strategy to attract nurses to assume formal nursing leadership roles. *Implications for Nursing Management*. Strategies to improve nurses' motivation to lead in a complex healthcare environment include improving nurses' active involvement in their organization, creating a collegial supportive and mentoring leadership culture, and improving transformative exchange by supporting career and educational advancement.

## 1. Introduction

Nursing leadership is a critical factor that influences healthcare quality, patient outcomes, and organizational success. Effective nurse leaders guide teams, create positive work environments, and contribute to increased job satisfaction among nurses [1]. The role of nurse leaders is particularly crucial in addressing contemporary healthcare challenges and ensuring the resilience of nursing teams [2, 3]. Nurses' involvement in leadership positions within the healthcare system is a dynamic and evolving aspect which is crucial to delivering quality patient care and contributing to the overall effectiveness of healthcare organizations [4]. Traditionally, nurses have been at the forefront of direct patient care. However, there is a growing recognition of the need for nurses' active participation in leadership roles to address the complexities of modern healthcare [5, 6]. The involvement of nurses in leadership positions contributes to improving decision-making processes, enhancing coordination among healthcare teams, and fostering positive work environments [7]. Furthermore, as healthcare systems face challenges such as nursing shortages and increased demands, the active engagement of nurses in leadership positions becomes even more critical [8, 9].

Previous research on motivation to lead in the healthcare sector highlights the turnover intention of current nurse leaders [10, 11] and a persistent shortage of nurses' willingness to step into formal leadership roles [12, 13]. This scarcity is a critical concern given the acknowledged importance of motivating nurse leaders in achieving organizational goals and improving patient outcomes [1]. Upon closer examination, organizational variables emerged as significant contributors to nurses' hesitancy in embracing formal leadership roles. Issues such as inadequate leadership preparation, limited growth opportunities, and unsupportive work conditions emerged as crucial determinants impacting motivation to lead [14, 15]. This underscores the role played by the organizational context in shaping individuals' motivation to assume leadership responsibilities.

Recent research by Labrague et al. [9] shows a positive correlation between nurse confidence and desire to lead when working with authentic leaders. This emphasizes the influential role of the leadership environment on motivation to lead. In Oman, nearly 70% of millennial nurses expressed an intention for career advancement in assuming nursing leadership responsibilities [16]. Currently, in Oman, there are 21,288 registered nurses and a significant proportion are expatriates [17]. Fostering a culture that encourages nurses to assume leadership roles becomes essential to build a resilient and adaptable nursing workforce [17]. Understanding the factors that motivate nurses to assume leadership responsibilities is a critical step in tailoring interventions and support systems that align with the unique challenges and dynamics of the Oman health system.

Although several studies investigated the factors that contribute to nurses' motivation to lead [9, 13, 14, 18], limited evidence exists on the influence of the leadership environment. Therefore, the aim of this study was to examine the influence of leadership environment on nurses' motivation to lead. The study hypothesis is that there is a significant relationship between leadership environment and nurses' motivation to engage in leadership roles. In addition, the study intended to determine whether there are particular aspects of the leadership environment that influence motivation to lead. A unique contribution of this study is the use of complexity leadership theory, which contributes to the limited empirical research in this area. It also facilitates a better understanding of the complex relationship between nurses' motivations to assume leadership roles and specific dimensions of the leadership environment.

## 2. Theoretical Framework

The current study was guided by the complexity leadership theory. Complexity science posits that a system is more aptly described as a complex adaptive system (CAS), where the structure is nonhierarchical and nonlinear [19, 20]. People, objects, or processes (known as agents) have widespread impact on the system and from the system outward. Leadership culture is the feature that makes the difference and determines the impact of agents [5, 21].

In a CAS, the leaders (sometimes nurse managers, directors, or chief nursing officers) create the space to work (known as containers) so that several outcomes can be achieved simultaneously. The process in a CAS is therefore nonlinear. The advantage of a nonlinear system is the allowance for self-organization that supports shared governance and decision making. In any level in a CAS, accountability particularly resides in shared power and decision making [21]. People, in a CAS, bring different perspectives and resources to relationship exchanges where each exchange has the potential to transform the other agent [22]. These exchanges support the emergence of new or unexpected ideas, structures, or patterns of work. People, when in relationship and involved in transformative exchanges, are also changed in the process of coevolution [21]. Most healthcare systems, within which nurses work, have the attributes of a CAS.

A person motivated to lead does so within a system or context. The leadership context or leadership environment is important to identify, as some of the aspects of that environment can be modified to foster nurses' feelings of support and, therefore, a desire to lead. According to the CAS, those aspects are self-organization, agents, shared governance and decision making, emergence, transformative exchanges, different perspectives, and coevolution [23].

**Figure 1:**
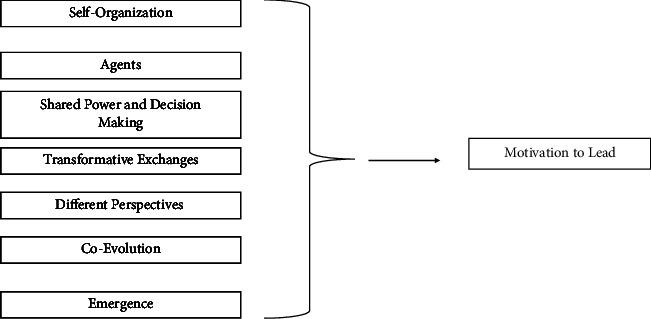
Theoretical framework.

The seven CAS aspects of the work environment, mentioned above, have relatedness to the constructs of motivation (assuming more or higher-order responsibilities and advancement in organization positions) and leadership (involvement in and influence in decision making). The framework shows a unidirectional impact (Figure 1). Motivation to lead occurs within the context or leadership environment. This is the same as nurse leaders setting the space/container for self-organization, engaging agents (in the process of motivation), and allowing transformative exchanges between agents with different perspectives. What follows is the emergence of coevolution that can also become shared power and decision making, hallmarks of leading. Since the research aim was to delineate the pertinent aspects of the leadership environment that are related to willingness to lead, the complexity leadership theory was chosen over other leadership theories or styles. The LENS as a measure will provide details of the environment that can be enhanced or mitigated in order for more nurses to be willing to lead. Other measures of leadership do not completely apply to the context or environment within which nurses lead.

## 3. Materials and Methods

### 3.1. Study Design and Setting

A cross-sectional design was used. Self-reported data were collected from staff nurses working in 15 acute care public and private hospitals in the Sultanate of Oman. The hospitals were distributed over different geographical areas of Oman, making the sample highly representative of the nursing workforce working in the acute care setting in Oman.

### 3.2. Study Sample

A convenience sampling technique was adopted to gather data from registered nurses working in public and private hospitals in the Sultanate of Oman. The use of convenience sampling might have created sampling bias. To reduce it, the study utilized a national sample by including hospitals from different governorates of Oman.

The inclusion criteria were registered staff nurses providing bedside care, holding a minimum diploma in nursing, being licensed, and having at least one year of experience in their current unit, and able to read and write English language. Unit managers were excluded from the study as they are in a leadership position, which negates the main purpose of the study. The estimated sample size of the study was 540 participants based on the G-Power priori sample size calculator [24]. A statistical power of 80%, 10 predictors, a small effect size, and a probability level of 0.05 were used to calculate this sample. The survey was distributed to 540 nurses, where a total of 435 nurses participated, resulting in an 80.6% response rate.

### 3.3. Study Instruments

Data were collected using a paper-based survey. All study instruments were administered in English. The study instruments consisted of three sections: sociodemographic information, the Leadership Environment Scale, and the Motivation to Lead Scale. The sociodemographic section includes questions about the age, sex, years of work experience, education level, nationality, and previous leadership experience and training.

The perceived leadership environment was measured using the 16-item Leadership Environment Scale (LENS) [23]. The scale assesses nurses' perceptions of nurses about seven domains of the leadership environment including (1) self-organization, which is defined as the creation of self-organizing systems (by individuals or groups) through feedback, self-reference, and effective information use; (2) agent, which is defined as a person, object, or process that has an impact on a system at a local and global level, and these agents provide insights into the structure of a complex adaptive system; (3) shared power and decision making, which are the key components of a self-organizing system that ideally occur at the point where actions are to be executed and accountability is expected; (4) emergence, which is defined as an unexpected idea, structure, or pattern that arises without any prior warning or preparation; (5) transformative exchanges, which refer to a series of interdependent connections within a complex adaptive system where information and resources are shared in such a way that each agent involved is altered as a result of the exchange; (6) different perspectives, which are defined as differences that affect both the work and relationships within a system; and (7) coevolution, which is defined as the mutual change that occurs between them when they are in relationship [23]. As one agent adapts and mirrors, another agent also changes. These domains were adopted from the key features of complexity leadership theory [21]. Responses were rated using a 4-point Likert scale in which 1 refers to strongly disagree and 4 refers to strongly agree. Higher scores indicate a more favorable perception of the leadership environment. The scale is valid and reliable with Cronbach's alpha of 0.91 [23]. In the current study sample, Cronbach's alpha was 0.81.

Nurses' motivation to engage in formal leadership roles was measured using the 3-item Motivation to Lead Scale (MTL) [25]. Responses were rated using a 5-point Likert scale in which 1 refers to strongly disagree and 5 refers to strongly agree. Higher scores indicate a greater motivation to engage in formal leadership roles. The scale is valid and reliable with Cronbach's alpha of 0.78 [16]. In the current study sample, Cronbach's alpha was 0.88.

All of the data were collected using a self-reported method. To reduce the possibility of the response bias, all data were collected anonymously with no identification.

### 3.4. Ethical Considerations and Data Collection Procedure

Prior to data collection, ethical approval was obtained from the Oman Ministry of Health Research and Studies Approval Committee (MoH/CSR/22/26226). The recruited hospitals also provided administrative permission to collect the data. Written informed consent was obtained from the study participants.

Participants received a package containing an information sheet, an informed consent form, a survey, and an envelope. This information sheet provided information about the study's aims, procedure, the time required to complete the survey, assurances of voluntary participation and confidentiality, and contact information for the principal investigator for additional clarifications or information if needed. To facilitate the deposit of completed surveys, drop boxes were located in nursing stations. Data were collected over five months, between August 2022 and February 2023.

### 3.5. Data Analysis

The Statistical Package for the Social Sciences (SPSS)® version 23 was used to analyze the data. Descriptive statistics of frequencies, standard deviations, and means were used to describe the study sample. Multivariate linear regression was used to examine the relationship between the leadership environment and nurses' motivation to lead, controlling for the sociodemographic and work characteristics of the participants. The significance level was established at *p* < 0.05. No missing data were reported and no sensitivity analyses were conducted.

## 4. Results

A total of 435 nurses participated in the study. The sample was predominantly female (87.4%), with an average age of 43.85 years (SD = 6.549) and 11.40 years of work experience (SD = 6.652). Of the 435 participants, more than half were expatriate (51.3%), and 56.1% held a diploma in nursing. The majority of the sample had no previous leadership experience or received leadership training (66.7% and 70.8%), respectively (Table 1).

### 4.1. Perception of Leadership Environment and Motivation to Lead

The mean scale value for LENS was 3.194 out of 4 (SD = 0.661). Across the seven leadership environment domains, nurses perceived emergence followed by the transformative exchange as the highest. Nurses perceived self-organization as the lowest available feature of their leadership environment in which nurses reported lack of time to participate actively in changing their work setting (Table 2).

The mean scale value for MTL was 3.208 out of 5 (SD = 0.467). The findings also revealed that most of the study participants (85.1%) reported a desire to advance their career and assume greater responsibility. Additionally, 83.5% indicated an intention to assume different responsibilities in their current roles and 83.5% intend to advance to positions that involve more decision making (Table 3).

### 4.2. Relationship between Leadership Environment and Motivation to Lead

A multivariate regression model was conducted to assess the relationship between nurses' perception of the current leadership environment and the reported motivation to engage in leadership roles controlling for age, years of experience, nationality, level of education, type of working unit and hospitals, previous leadership training, and experiences (Table 4). After testing for multicollinearity, no evidence of multicollinearity was observed.

The findings demonstrated a significant relationship between leadership environment and nurses' motivation to engage in leadership roles controlling for other covariates, supporting the study hypothesis. In addition, the study intended to determine whether there are particular aspects of the leadership environment that influence motivation to lead. Findings demonstrated that self-organization (*β* = 0.185, *p*=0.001, CI = 0.086–0.378), agents (*β* = 0.221, *p*=0.002, CI = 0.124–0.474), and transformative exchange (*β* = 0.100, *p*=0.037, CI = 0.101–0.142) were specific elements of the leadership environment associated with higher motivation to engage in leadership. The overall *R*^2^ of the model was 0.38, which reflects that the model was successful in explaining 38% of the variance in nurses' motivation to lead the score.

## 5. Discussion

This study examined the relationship between leadership environment and nurses' motivation to lead. The mean score obtained on the LENS suggests that nurses perceive their leadership environment positively. In other words, they likely view their leadership as supportive, effective, and conducive to their professional growth and well-being. This result is encouraging given that a favorable leadership environment is critical for the overall success and well-being of an organization [26]. When leaders create an atmosphere of trust, respect, and support, employees are more likely to feel satisfied and engaged in their job [27]. Moreover, a favorable leadership environment fosters a culture of collaboration and open communication, where ideas are freely exchanged, and innovation is encouraged [28, 29]. This, in turn, leads to higher levels of productivity and better organizational outcomes [30].

The mean score on the MTL, which exceeds the midpoint, suggests a strong desire or motivation among people to pursue formal nursing leadership roles in the future. Across the MTL items, 85.1% of the participants expressed a desire to advance in the nursing career and assume more responsibilities. This aligns with Labrague et al.'s [16] findings where 70% of nurses shared a similar aspiration for career advancement. This result implies that there is a notable readiness and eagerness among nurses to embrace formal nursing leadership roles, potentially contributing to the cultivation of future nursing leaders and the advancement of nursing practice and healthcare delivery. The overarching endorsement of career advancement among nurses in the present study emphasized the necessity of structural empowerment for upward mobility in leadership and highlighted the crucial role of the work environment in fostering nurses' willingness to assume leadership roles.

The results of the multiple regression analysis showed a positive relationship between leadership environment at work and nurses' motivation to lead. To our knowledge, this study was the first to explore the specific features within the leadership environment that strongly influence nurses' motivation to undertake leadership roles. Hence, this study provided the necessary information to optimize organizational efforts and drive meaningful improvements in leadership development. Nevertheless, the results from the current study suggested that when nurses perceive their work environment as supportive, conducive to growth and development, and characterized by effective leadership, they are more likely to be motivated to pursue leadership roles in the nursing profession. In other words, a more favorable and empowering work environment, where leadership is perceived positively, can serve as a catalyst for fostering nurses' aspirations and intentions to take on leadership responsibilities. Previous studies have shown that with a desirable work environment, nurses are provided with management and leadership advancement, engagement, coaching, mentoring, leadership modeling, and adequate structural resources, which all contributed to increase their willingness to engage in leadership tasks [9, 11, 31]. Positive work environments can increase the intention of nurses to lead, as well as enhance their professional development. This finding was reported in previous research that suggested that a favorable work environment is positively associated with the leadership career advancement [9, 13, 32]. Overall, this finding underscores the importance of cultivating a nurturing and supportive organizational culture. This can inspire nurses to develop their leadership potential, which ultimately contributes to the advancement of nursing practice and the delivery of quality patient care.

A significant contribution of the present study lies in its ability to link the results to Ross et al.'s conceptual framework, framing the results through the seven dimensions of the LENS. The framework was proposed to describe the leadership environment experienced by nurses in their practice settings. While the LENS respondents were direct care nurses from Oregon in the USA, the current study respondents were direct care nurses from Oman. Oregon nurses were on average 10 years older and there was a 10% higher number of nurses with baccalaureate degrees [23]. However, the percentage of nurse respondents in both countries who reported they were female was the same. These similarities made drawing conclusions about the work environment and its effect on the leadership environment very sensible. In fact, the findings of the current study support the notion that, regardless of the cultural difference between different countries, the workplace environment is still a critical factor that influences the leadership environment among direct care nurses. It is important to acknowledge that the leadership environment between the two countries is different. For example, the findings of the current study suggested that nurses identified emergence, closely followed by transformative exchanges, as the highest features of the leadership environment. This differs from the results from LENS study in Oregon, where the highest endorsement was for coevolution, emphasizing mutual benefit in working with others.

The specific domains of the leadership environment that significantly influenced nurses' motivation to lead were self-organization, agents, and transformative exchanges. These domains are malleable and can be influenced by relationships and system supports, as indicated by the two-factor solution proposed by Ross et al. [23]. Self-organization is oriented towards the individual and how they can foster relationships within their organization. Nurses in the current study rated self-organization as the least obvious feature of the leadership environment. A similar finding was reported by Ross et al. [23]. According to the complexity theory, self-organizing systems emerge through the ability to be self-directed and effective utilization of available resources and information [5, 23]. This indicates the need to provide nurses with adequate knowledge, skills, resources, and authority to be actively involved in decision making and organizational change.

Agents are codependent on their relationship with each other, people, processes, and perspectives, specifically mentorship, understanding of different perspectives, and modeling leadership, and they can influence systems at a distance, such as seen in the butterfly effect (a hallmark of the CAS). Transformative exchanges are the currency that the health system uses to influence locally and at a distance. Transformative exchanges are used in self-organization between agents within the system. These exchanges show nurses the support they have from their supervisor and the system, as well as how the system supports their educational advancement into potential leadership roles [21]. In order to improve the transformative exchange within healthcare settings, it is important to foster a culture of continuous learning and mentorship, by current nurse leaders. Encouraging ongoing professional development and providing mentoring opportunities can contribute significantly to the growth of potential leaders.

Motivation to lead is a multifaceted construct influenced by a variety of factors [14, 15]. Given that only 38% of the variance in motivation to lead was explained by the leadership environment, there may be other significant elements contributing to this outcome. High workloads and inadequate staffing are crucial factors that can lead to burnout, work dissatisfaction, and reduced motivation to take on additional leadership responsibilities [13, 16, 33, 34]. Educational background and professional development opportunities also play a significant role; nurses with higher education levels and access to continuous learning may be more inclined to pursue leadership roles [16]. Personal characteristics, such as leadership self-efficacy, career aspirations, and individual interest in leadership, further impact motivation [7, 16, 35]. Moreover, the presence of structural elements such as professional advancement opportunities, robust leadership and managerial support, and sufficient hospital resources may profoundly influence nurses' confidence in their leadership abilities and, consequently, their inclination to assume leadership roles [7, 15, 16]. These confounding factors suggest that a multifaceted approach is necessary to fully understand and enhance nurses' motivation to lead. Hence, future research should be conducted to take these elements into account and provide a more comprehensive understanding of what drives nurses to assume leadership roles.

### 5.1. Implications for Nursing Management

The most important implications of this study are that leaders should be aware of the importance of the leadership environment to front-line leaders, as well as their capacity to influence and create such environment. According to this study, emergence and transformational exchanges dominate the leadership environment, indicating that establishing a positive team culture is key to creating a desirable work environment. In addition, by investing in succession planning and mentorship programs, nurse leaders can ensure a smooth transition of leadership roles and sustain the continuity of effective leadership within the nursing workforce [29]. The findings of this study point to the importance of changing the leadership environment. Even though previous studies by Cziraki et al. [7] and Mascia et al. [25] found that self-efficacy was a significant factor in MTL, the environment within which leaders develop and lead may trump self-efficacy or any demographic factors. In the leadership environment, agents, self-organization, and transformative exchanges are the strongest domains that can influence motivation to lead.

The other implications that can be linked to the findings of the current study are that nurse managers are to take the lead in changing the environment within their sphere of influence and setting the container or creating the space, so nurses not only cope with stress and change but also can fulfil their potential as leaders. To achieve this, nurse leaders are strongly recommended to implement the following strategies. First, as an agent, the nurse leaders are required to create the space and set the container within which all the work of developing new nurse leaders will occur. Second, nurse leaders identify candidates for leadership development who are known to have diverse perspectives. Third, create, in conjunction with the leadership candidate, pertinent resources to support the development of leadership skills. Fourth, provide opportunities for self-organization and transformative exchanges while staying in relationship.

Lastly, it is important to periodically assess the leadership environment, especially before any planned changes within the work environment. This baseline assessment can provide better insight into the change in the work environment and its relationship to the leadership environment at the unit and institutional levels.

### 5.2. Study Limitations and Future Research

Because the study used a cross-sectional design, the causal relationship between leadership environment and nurses' motivation to lead was limited. Additionally, the sample included nurses working in acute care hospitals; therefore, the generalizability of the study findings to other types of hospitals could be limited. Future study utilizing a longitudinal design including nurses from diverse hospitals such as general primary, tertiary, and acute care hospitals, assessing nurses' perception of their leadership environment and motivation to lead are recommended. Suggestions for future research also include conducting a longitudinal interventional study measuring the LENS prior to educational enhancement on leadership development and mentorship and three to six months after the intervention.

## 6. Conclusions

The study findings highlight the critical need to foster a nurturing and supportive organizational culture that motivates nurses to explore and expand their leadership capabilities. By doing so, organizations can significantly advance nursing practice and enhance the quality of patient care they provide. Recognizing aspects of self-organization, agent dynamics, and transformative interactions within the healthcare system presents a strategic approach to nurturing nurses' motivation to assume leadership roles, fostering a positive work culture, and improving the overall leadership environment in healthcare settings. While this study was conducted specifically within the context of Oman, the implications of fostering a supportive nurse practice environment are broadly relevant to healthcare systems globally. Healthcare organizations worldwide can benefit from these insights by investing in leadership development and creating a culture that supports nurses, ultimately leading to improved patient outcomes and enhanced nursing practice.

## Figures and Tables

**Table 1:** Sample characteristics (*N* = 435).

Variables	Mean	SD
Age	34.853	6.549
Years of experience	11.396	6.652

	*n*	%

Gender		
Male	55	12.6
Female	380	87.4
Nationality		
Indigenes (local)	212	48.7
Expatriate	223	51.3
Highest education level		
Diploma	244	56.1
Baccalaureate	178	40.9
Master	13	3.0
Type of working unit		
Medical/surgical	134	30.8
Critical care	97	22.3
Neonatal and pediatric	42	9.7
Maternity	103	23.7
Operating room	34	7.8
Others^*∗*^	25	5.7
Hospital teaching status		
Teaching	216	49.7
Nonteaching	219	50.3
Hospital type		
Public	362	83.2
Private	73	16.8
Previous leadership experience		
No	290	66.7
Yes	145	33.3
Previous leadership training		
No	308	70.8
Yes, and it was adequate	96	22.1
Yes, but it was not adequate	31	7.1

^*∗*^Included nurses working in multispecialty units, outpatient clinics, and cath lab.

**Table 2:** Descriptive summary and responses on Leadership Environment Scale.

Item	Mean	SD
Leadership Environment Scale^*∗*^	3.194	0.661
Self-organization	3.135	0.503
I have time to actively participate in efforts to change practice in my work setting	3.064	0.599
Nursing determines its own policies and procedures	3.206	0.545
Agents	3.173	0.473
Nurse colleagues are demonstrating leadership in nursing practice	3.206	0.537
In my work setting mentorship is available to me for my day-to-day work and/or career	3.172	0.564
People I work with appreciate my perspectives, even though these may be different from their own	3.142	0.613
Shared power and decision making	3.217	0.516
Nurses in my workplace have good working relationship with other professionals	3.255	0.561
The administration listens and responds to employee concerns	3.183	0.598
Emergence	3.309	1.155
By working as a group, we achieve more than any one of us alone	3.264	0.548
I engage in leadership in my work setting	3.211	0.630
Transformative exchange	3.231	0.847
The person I report to works to have a good relationship with me	3.225	0.625
My organization supports me in advancing my education	3.200	0.660
My organization supports me in advancing my career within our system	3.179	0.610
Different perspectives	3.202	0.509
Other disciplines value the work of nursing	3.225	0.551
Innovative ideas about patient care are supported	3.179	0.563
Coevolution	3.192	0.527
When working with other people our relationships mutually benefit each other	3.227	0.572
I take time to reflect on my work and career	3.156	0.582

^*∗*^Scale composite score.

**Table 3:** Response on the Motivation to Lead Scale.

MTL items	Strongly disagree	Disagree	Neither	Agree	Strongly agree
	*n* (%)	*n* (%)	*n* (%)	*n* (%)	*n* (%)
(1) I desire career advancement to assume more responsibility	5 (1.1%)	11 (2.5%)	49 (11.3%)	277 (63.7%)	93 (21.4%)
(2) I would like to take on different responsibilities compared to those my job position requires	1 (0.2%)	22 (5.1%)	49 (11.3%)	274 (63%)	89 (20.5%)
(3) I would like to advance in organizational positions that require greater involvement and influence in organizational decision-making processes	1 (0.2%)	19 (4.4%)	52 (12%)	263 (60.5%)	100 (23%)

**Table 4:** Multiple regression on the relationship between leadership environment and nurses' motivation to engage in leadership roles.

Variables	MTL				
	*B*	SE	*β*	*t*	*p* value
(Constant)	1.422	0.331		4.290	<0.001
Leadership environment					
Self-organization	0.238	0.072	0.185	3.322	**0.001**
Agents	0.283	0.087	0.221	3.031	**0.002**
Transformative exchange	0.075	0.036	0.100	2.051	**0.037**
Different perspectives	0.080	0.078	0.065	1.029	0.304
Coevolution	0.148	0.076	0.122	1.945	0.066
Shared power and decision making	0.073	0.077	0.057	0.945	0.345
Emergence	0.006	0.024	0.011	0.257	0.798
Sociodemographic characteristics					
Age	0.006	0.024	0.011	0.254	0.800
Men versus women	0.035	0.058	0.027	0.596	0.551
Years of experience	0.001	0.008	0.009	0.110	0.913
Expatriate versus indigene nurses	−0.011	0.008	−0.111	−1.295	0.196
Having leadership experience versus not having	0.019	0.079	0.010	0.241	0.810
Having leadership training versus not	0.022	0.066	0.016	0.338	0.735
Education (reference: diploma)					
BSN degree	0.055	0.048	0.053	1.143	0.254
MSN degree	−0.036	0.062	−0.027	−0.578	0.564
Private versus public hospital	0.043	0.092	0.026	0.468	0.640
Teaching versus nonteaching hospital	0.055	0.054	0.043	1.009	0.314

MTL: Motivation to Lead Scale. *p* < 0.05.

## Data Availability

The data used to support the findings of this study are available from the corresponding author upon request.
